# *Staphylococcus saprophyticus* From Clinical and Environmental Origins Have Distinct Biofilm Composition

**DOI:** 10.3389/fmicb.2021.663768

**Published:** 2021-06-07

**Authors:** Opeyemi U. Lawal, Marta Barata, Maria J. Fraqueza, Peder Worning, Mette D. Bartels, Luisa Goncalves, Paulo Paixão, Elsa Goncalves, Cristina Toscano, Joanna Empel, Malgorzata Urbaś, Maria A. Domiìnguez, Henrik Westh, Hermínia de Lencastre, Maria Miragaia

**Affiliations:** ^1^Laboratory of Bacterial Evolution and Molecular Epidemiology, Instituto de Tecnologia Química e Biológica António Xavier, Universidade NOVA de Lisboa, Oeiras, Portugal; ^2^Laboratory of Molecular Genetics, Instituto de Tecnologia Química e Biológica António Xavier, Universidade NOVA de Lisboa, Oeiras, Portugal; ^3^Centre for Interdisciplinary Research in Animal Health, Faculdade de Medicina Veterinária, Universidade de Lisboa, Lisbon, Portugal; ^4^Department of Clinical Microbiology, Hvidovre University Hospital, Hvidovre, Denmark; ^5^SAMS Hospital, Lisbon, Portugal; ^6^Hospital da Luz, Lisbon, Portugal; ^7^Hospital Egas Moniz, Lisbon, Portugal; ^8^Department of Epidemiology and Clinical Microbiology, Narodowy Instytut Leków, Warszawa, Poland; ^9^Hospital Universitari de Bellvitge, Barcelona, Spain; ^10^Institute of Clinical Medicine, Faculty of Health Sciences, University of Copenhagen, Copenhagen, Denmark; ^11^The Laboratory of Microbiology and Infectious Diseases, The Rockefeller University, New York, NY, United States

**Keywords:** *Staphylococcus saprophyticus*, evolution, pan-GWAS, WGS, biofilm structure, *ica* cluster, urinary tract infection

## Abstract

Biofilm formation has been shown to be critical to the success of uropathogens. Although *Staphylococcus saprophyticus* is a common cause of urinary tract infections, its biofilm production capacity, composition, genetic basis, and origin are poorly understood. We investigated biofilm formation in a large and diverse collection of *S. saprophyticus* (*n* = 422). Biofilm matrix composition was assessed in representative strains (*n* = 63) belonging to two main *S. saprophyticus* lineages (G and S) recovered from human infection, colonization, and food-related environment using biofilm detachment approach. To identify factors that could be associated with biofilm formation and structure variation, we used a pangenome-wide association study approach. Almost all the isolates (91%; *n* = 384/422) produced biofilm. Among the 63 representative strains, we identified eight biofilm matrix phenotypes, but the most common were composed of protein or protein–extracellular DNA (eDNA)–polysaccharides (38%, 24/63 each). Biofilms containing protein–eDNA–polysaccharides were linked to lineage G and environmental isolates, whereas protein-based biofilms were produced by lineage S and infection isolates (*p* < 0.05). Putative biofilm-associated genes, namely, *aas*, *atl*, *ebpS*, *uafA*, *sasF*, *sasD*, *sdrH*, *splE*, *sdrE*, *sdrC*, *sraP*, and *ica* genes, were found with different frequencies (3–100%), but there was no correlation between their presence and biofilm production or matrix types. Notably, *icaC_1* was ubiquitous in the collection, while *icaR* was lineage G-associated, and only four strains carried a complete *ica* gene cluster (*icaADBCR*) except one that was without *icaR*. We provided evidence, using a comparative genomic approach, that the complete *icaADBCR* cluster was acquired multiple times by *S. saprophyticus* and originated from other coagulase-negative staphylococci. Overall, the composition of *S. saprophyticus* biofilms was distinct in environmental and clinical isolates, suggesting that modulation of biofilm structure could be a key step in the pathogenicity of these bacteria. Moreover, biofilm production in *S. saprophyticus* is *ica*-independent, and the complete *icaADBCR* was acquired from other staphylococci.

## Introduction

*Staphylococcus saprophyticus* is a uropathogen associated with 10–20% of urinary tract infection (UTI) in sexually active young women worldwide (Raz et al., 2005; Kline and Lewis, 2016). Possible complications such as acute pyelonephritis, urethritis (Hovelius et al., 1984), and endocarditis (Garduño et al., 2005; Choi et al., 2006), especially in immunocompromised individuals, have been documented. *S. saprophyticus* is a frequent colonizer of the human gastrointestinal tract, cervix, urethra, vagina, perineum, and rectum (Latham et al., 1983; Rupp et al., 1992). Also, it colonizes the gut and skin of food-producing animals (Hedman et al., 1993), which could serve as a source of contamination of food-related environments.

The success of *S. saprophyticus* as a uropathogen is due to its ability to survive in harsh and toxic conditions, which is provided by the accumulation of genetic determinants encoding high resistance to heavy metals (Lawal et al., 2021b) and detoxification of uric acid and D-serine (Gatermann and Marre, 1989; Korte-Berwanger et al., 2013). Moreover, *S. saprophyticus* pathogenicity has been described to be associated with its capacity to adhere to uroepithelial cells promoted by adhesins, surface proteins, and biofilm production (Kuroda et al., 2005; Martins et al., 2019).

Previous studies mainly done on uropathogenic *Escherichia coli* have shown that biofilm is an important pathogenicity factor in either medical device-associated UTI (Jacobsen et al., 2008) or cystitis (Anderson et al., 2003; Rosen et al., 2007; Blango and Mulvey, 2010). In particular, it was demonstrated in a mouse model of cystitis that *E. coli* and *Klebsiella pneumoniae* can exist in biofilm-like large aggregates of bacteria (pods or intracellular bacterial communities) in the bladder epithelial cells, a phenomenon that was suggested to be responsible for recurrent cystitis (Anderson et al., 2003; Rosen et al., 2007; Blango and Mulvey, 2010). The importance of biofilm for UTI was additionally shown during occurrence of urinary stones/calculi by urease-producing bacteria (Norman and Stamey, 1971; McLean and Nickel, 1994). Regarding *S. saprophyticus*, the role of biofilm on pathogenesis was mainly evidenced by the occurrence of medical device-associated UTI (Hovelius et al., 1984; Choi et al., 2006; Magarifuchi et al., 2015), but it remains to be demonstrated whether biofilms formed by these bacteria are also implicated in cystitis or urinary stones.

Biofilms are organized bacterial cell communities contained in an extracellular matrix that mediate adherence to abiotic and biotic surfaces (Heilmann et al., 2003; Izano et al., 2008; Tojo et al., 2009; Fagerlund et al., 2016). Biofilms play a significant role in an array of infections, namely, medical device associated, valve endocarditis, and UTI (O’Gara, 2007; Becker et al., 2014). Bacterial cells within the biofilm matrix exhibit phenotypic characteristics different from those of planktonic or free-living cells (Martins et al., 2019). For instance, free-living bacteria cells that are susceptible to antibiotics sometimes become resistant or tolerant to such antibiotics or other antimicrobial agents in the matrix (Martins et al., 2019). In fact, *S. saprophyticus* biofilms were reported to be resistant to antibiotics used in the empirical treatment of UTI and to biocides used for decontamination because of the protective function of the biofilm against the action of these agents (Fagerlund et al., 2016; Martins et al., 2019). Additionally, biofilms constitute effective barriers against host-immune evasion and low urine pH (Becker et al., 2014; Heilmann et al., 2019). Biofilm could also be a hotspot for horizontal gene transfer and a risk for development and dissemination of multidrug-resistant strains (Baker-Austin et al., 2006; Fagerlund et al., 2016; Martins et al., 2019).

The composition of the biofilm matrix could be different between species and from strain to strain (Fagerlund et al., 2016), but the biofilm matrix is essentially composed of bacterial cells embedded in polysaccharides, extracellular DNA (eDNA), and proteins. In staphylococcal biofilms, polysaccharide intercellular adhesin (PIA) is one of the main components. PIA is a homoglycan with beta-1,6-linked *N*-acetylglucosamine residues and de-*N*-acetylated amino groups in its composition (Mack et al., 2016). The synthesis of PIA in biofilm formation is mediated by an operon (*icaADBCR*). This comprises the *N*-acetylglucosamine transferase *icaA* that synthetizes PIA oligomers from UDP-*N*-acetylglucosamine and the product of *icaD*, which gives optimal efficiency to *icaA* (Arciola et al., 2015). The *icaB* encodes *N*-deacetylase and is involved in the partial deacetylation of PIA. The product of *icaC* is involved in the export of the polysaccharide, while *icaR* is the negative transcriptional regulator of the operon (Rohde et al., 2010; Arciola et al., 2015).

Extracellular DNA has been described in staphylococcal biofilms from *Staphylococcus aureus* (Eckhart et al., 2007; Izano et al., 2008) and *Staphylococcus epidermidis* (Qin et al., 2007; Izano et al., 2008) and was described to be part of the biofilm of two clinical strains of *S. saprophyticus* (Soumya et al., 2017). Staphylococcal biofilms are often additionally composed of surface-associated and cell wall-anchored proteins such as microbial surface components recognizing adhesive matrix molecules, which are essential for different stages of attachment to surfaces and biofilm accumulation (Jönsson et al., 1991; Patti et al., 1994). Other commonly found proteins associated with proteinaceous biofilm in staphylococci are autolysins/adhesins such as AtlE, Aap in *S. epidermidis*, and Bap in *S. aureus* (Heilmann et al., 1997; Cucarella et al., 2001; Hirschhausen et al., 2012). In spite of the clinical relevance of *S. saprophyticus* biofilms, its composition remains unclear.

A recent study showed that *S. saprophyticus* causing UTI in humans belonged to two major clonal lineages (G and S) that originated in food/production animals and humans, respectively, (Lawal et al., 2021a). However, it is still unknown if the mechanisms of disease caused by the two lineages are related.

In this study, we aimed to explore the heterogeneity in matrix composition of biofilms produced by *S. saprophyticus* and to explore how biofilm phenotypes are distributed in the population and how they correlate with genetic content.

## Materials and Methods

### Ethical Considerations

The human isolates were recovered as part of the routine clinical diagnostic testing; ethical approval and informed consent were not required. All data were handled anonymously. Sample collection was in accordance with the European Parliament and Council decision for the epidemiological surveillance and control of communicable disease in the European community^1^. Slaughterhouse samples were part of the routine control practices for evaluation of good hygiene practices and programs to assure meat safety (CE No. 853/2004).

### Bacterial Collection

We assembled a large collection of 422 *Staphylococcus saprophyticus* isolates recovered in seven countries from human infection and colonization as well as food-related environment between 1997 and 2017 (Supplementary Table 1). Out of a total number of biofilm producers (*n* = 384), we selected 63 strains with high biofilm production representing the different phylogenetic clusters identified when isolates were studied by single-nucleotide polymorphism (SNP) analysis (Lawal et al., 2021a). The selected isolates comprised isolates from both clonal lineages G (*n* = 42) and S (*n* = 21; Supplementary Figure 1). Selected strains were recovered from human infection and colonization (*n* = 47) and food-related environment (*n* = 16; Supplementary Table 2).

### Biofilm Formation Assay

We assessed the biofilm formation capacity in 422 *S. saprophyticus* using the modified polystyrene microtiter plates in a static condition as previously described (Stepanović et al., 2000; Stephanovic et al., 2007). Briefly, a colony from an overnight culture was suspended in tryptic soy broth (TSB) and grown overnight at 37°C with aeration. The culture was adjusted to 0.5 McFarland standards with TSB supplemented with 1% glucose (w/v; BDH, England; TSBsG); and each suspension was inoculated onto 96-well microtiter plates (Corning Inc., United States) and incubated at 37°C for 18 h. The free-floating planktonic bacteria from each well were removed and washed (4×) with sterile distilled water. Attached cells were heat fixed at 60°C for 60 min and stained with 0.06% crystal violet; and excess dye was removed by washing (4×) with sterile deionized water. The plates were air-dried at room temperature. Biofilm produced by each isolate was quantified by adding 30% acetic acid to each well, measured for absorbance at OD_595 *nm*_, and classified as described below (Stephanovic et al., 2007). The assay was done in triplicates. *S. epidermidis* RP62A and TSB were used as positive and negative controls, respectively.

### Classification of Biofilm Production

The method of Stephanovic et al. (2007) was employed to classify the biofilm production. Briefly, the average OD_595 *nm*_ of the four blank wells (TSB only) was calculated, and the ODc was obtained by applying the following formula: ODc = AverageOD_595 *nm*_^*blank*^ + 4 ^∗^ Standard Deviation^*blank*^. The final optical density (OD) value of an isolate was expressed as average OD value of the strain less ODc. Biofilm formation for each test strain was classified as follows: OD ≤ ODc = no biofilm produced; ODc < OD ≤ 2 × ODc = weak biofilm producer; 2 × ODc < OD ≤ 4 × ODc = moderate biofilm producer; and 4 × ODc < OD = strong biofilm producer.

### Biofilm Detachment Assay

We used three biofilm-detaching agents previously described (Fagerlund et al., 2016; Sugimoto et al., 2018; Tasse et al., 2018), namely, Proteinase K (100 μg/ml, Sigma-Aldrich, St. Louis, United States), DNAse I (100 μg/ml, Sigma-Aldrich, St. Louis, United States), and sodium periodate (50 mM, Sigma-Aldrich, St. Louis, United States; prepared in sodium acetate buffer), which disperse biofilm matrix composed of protein, eDNA and polysaccharide, respectively. Biofilms were grown in microtiter plates in TSBsG for 18 h, as described above. The planktonic cells were removed, and the plates were washed with distilled water (4×). The disruptor was added to each well and incubated for 2 h at 37°C. For the control wells, only buffer was added instead. The suspensions were removed; the plates were washed, heat fixed, and stained with crystal violet as described above. Biofilm remaining after treatment with disruptors was determined by comparing the test assays with their respective control. The main component of the biofilms produced by each representative isolate was assessed and classified as described below. All assays were done in triplicates.

### Biofilm Composition and Definition of Biofilm Types

To determine the relative biofilm composition, we compared the biofilm biomass of each strain after disruption with its corresponding control (without disruptors) expressed in percentage (%), as previously described (Kogan et al., 2006; Fagerlund et al., 2016). Isolates with >70% reduction in biofilm after treatment with specific biofilm detaching agents were interpreted to be composed of the component targeted by the disruptor (Fagerlund et al., 2016); isolates with 30–70% reduction in biofilm after disruption were classified as partially composed of these components; and isolates with <30% biofilm reduction after disruption were considered as not containing the component (Kogan et al., 2006; Fagerlund et al., 2016).

### Whole-Genome Sequencing and Assembly

Paired-end sequence reads produced on an Illumina MiSeq with an average coverage of 103 per genome reported in Lawal et al. (2021a) with the accession number PRJNA604222 were retrieved from sequence read archives. Low Q-score ends (Q < 20) were trimmed of the Illumina reads using Trimmomatic v0.36 (Bolger et al., 2014). Reads were assembled using SPAdes v3.11.1 (Bankevich et al., 2012). QUAST v5.1 (Gurevich et al., 2013) was used to evaluate the quality of assemblies. All contigs with <200-bp size were removed.

### Phylogeny Reconstruction and Comparison

Single-nucleotide polymorphism-based phylogeny of all *S. saprophyticus* isolates in the collection and the representative strains was done separately using CSIPhylogeny v1.4 (Kaas et al., 2014) with the default parameters. Maximum likelihood trees were reconstructed using RAxML v8.2.12 (Stamatakis, 2014). The general time reversible model was performed with 100 bootstrap resampling for node support. Phylogenetic trees were re-rooted midpoint and visualized using web-based tool microreact (Argimón et al., 2016).

### Genome Annotation and Pangenome Construction

Genomes were annotated using Prokka v1.14.6 (Seemann, 2014). The pangenomes of the representative strains (*n* = 63) and those of the entire collection (*n* = 422) were defined using Roary v3.13.0 (Page et al., 2015) with 85% BLASTp homologues clustering with split paralogues. The accessory genomes were defined as the pan minus the core genome. Pangenome-wide association study (pan-GWAS) approach was used to determine the association between genomic, demographic, and phenotypic data using Scoary v1.6.16 (Brynildsrud et al., 2016). Genes with a Benjamini–Hochberg *p* value < 0.05 and odds ratio > 1, with no duplicated function in the pangenome, were considered.

### Comparative Genomic Analysis

The nucleotide sequences of genes of interest found in the collection were compared using blast (blastn) analysis (Altschul et al., 1997, 2005). Contigs were reordered with MAUVE (Darling et al., 2004) using *S. saprophyticus* American Type Culture Collection (ATCC) 15305 (AP008934.1) as reference. Gene environment and synteny of specific loci were compared and visualized using Artemis v17.0.1 (Carver et al., 2012) and Easyfig (Sullivan et al., 2011).

### Statistical Analysis

The mean and standard error values of technical and biological replicates of each strain were calculated. The statistical significance of differences between the control and the respective disruption assay for each strain was done through unpaired Student’s *t*-test. Chi square was used to test the significance of the link between *S. saprophyticus* biofilm phenotypes of strains from different lineages and origin. Statistical analyses were performed with GraphPad Prism v6.0 (GraphPad Software, Inc., San Diego, CA, United States).

## Results

### The Great Majority of *Staphylococcus saprophyticus* Isolates Produced Biofilm

All *S. saprophyticus* isolates (*n* = 422) recovered from human colonization and infection including UTI and food-related environment were assessed for their ability to produce biofilm. A great majority (91%; 384/422, OD_595 *nm*_ > 0.2) produced biofilm, among which 91% (*n* = 349/384) were strong biofilm producers, and 5% each (*n* = 18/384) were moderate and weak biofilm producers. Among the weak (*n* = 18) and non-biofilm producers (*n* = 36) in this collection, 83% (*n* = 15/18) and 64% (23/36), respectively, were recovered from human infection and dispersed in clonal lineage G.

### Biofilm Composition Is Highly Diverse in *Staphylococcus saprophyticus*

Biofilm matrix phenotype in 63 representative *S. saprophyticus* isolates (Supplementary Figure 1) was determined using biofilm detachment assay. Our results showed that proteinase K, when compared with the controls, detached > 75% of the biofilm matrix formed in almost all the isolates (98%; 62/63) in this collection. The treatment of isolates’ biofilm matrix with DNAse and sodium periodate showed that in 54% (34/63) and 46% (29/63) of the isolates, respectively, >70% of the matrix was detached (Supplementary Figure 2), while a partial detachment (30–70% biofilm detached) was observed in 35% (22/63) and 41% (26/63), respectively, (Supplementary Figure 2). Considering that the amount of biofilm detached is directly correlated to the amount of the targeted biofilm component, almost all the *S. saprophyticus* tested produced biofilms composed of similar amounts of proteins, while the content in eDNA and polysaccharide varied from strain to strain.

**FIGURE 1:**
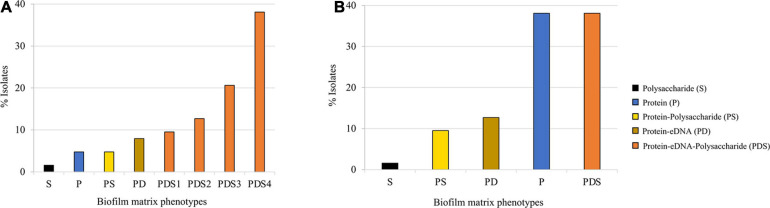
Quantitative classification of preformed biofilm in 63 *Staphylococcus saprophyticus* strains based on matrix phenotypes. **(A)** Activity of biofilm-degrading agents, namely, proteinase K, DNase, and sodium periodate, was assessed on biofilm produced. Isolates with >70% biofilm reduction after treatment with specific biofilm detaching agents were interpreted to be composed of the component targeted by the disruptor, while 30–70% or <30% biofilm reduction after disruption were expressed as partially composed or not composed of the targeted biofilm components, respectively. S, polysaccharide; P, protein; PS, protein–polysaccharide; PD, protein–partial eDNA; PDS1, protein–polysaccharide–partial eDNA; PDS2, protein–partial polysaccharide–eDNA; PDS3, protein–eDNA–partial polysaccharide; and PDS4, protein–polysaccharide–eDNA. **(B)** Biofilm matrix phenotypes were categorized based on the major component such that components that were partially present (30–70% biofilm detached) in the biofilm produced by isolates were excluded. S, polysaccharide; PS, protein–polysaccharide; PD, protein–partial eDNA; P, protein; and PDS, protein–polysaccharide–eDNA. Assays were carried out in triplicates. All assays were carried out in triplicates.

We classified the observed phenotypes into groups based on the % of reduction in biofilm biomass after detachment in comparison with the respective control. Based on this classification, we found five major different biofilm matrix types in *S. saprophyticus* population. The great majority (81%; 51/63) of the isolates produced biofilm composed of protein–eDNA–polysaccharide (PDS). The remaining isolates (<20%) produced biofilms composed of protein–eDNA (PD), protein–polysaccharide (PS), protein only (P), and polysaccharides only (S; Figure 1A). Within the PDS-based biofilm, the quantity of eDNA and polysaccharides varied within the biofilm (PDS1–PDS4; Figure 1A), providing an additional layer of heterogeneity.

Next, we categorized the biofilm matrix phenotypes based on the major component such that components that were partially present (30–70% biofilm detached) in the biofilm produced by isolates were excluded. Based on this qualitative classification, 38% (24/63, each) of the isolates produced biofilm mainly composed of protein or PDSs, 13% (8/63) of PD, 10% (6/63) of PSs, and 2% (1/63) of S (Figure 1B).

### Biofilm Matrix Types in *Staphylococcus saprophyticus* Are Associated With the Genetic Background and the Source of Isolates

**FIGURE 2:**
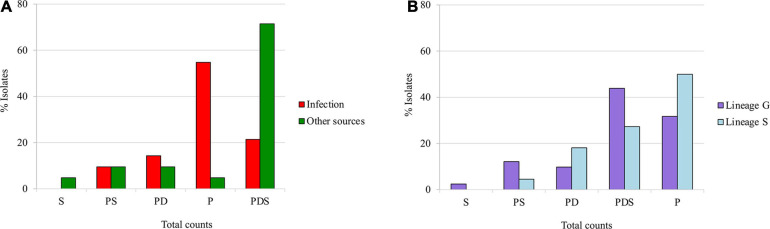
Classification of the biofilm matrix phenotypes produced by *Staphylococcus saprophyticus* based on the **(A)** source and **(B)** genetic lineages of isolates. Associations of biofilm matrix phenotypes with *S. saprophyticus* isolates belonging to two different genetic lineages and recovered from different sources were tested using chi square at *p* < 0.05. The proportions of isolates of infection and colonization **(A)** and lineage G and S **(B)** that have a specific biofilm phenotype are shown.

*Staphylococcus saprophyticus* belonging to different genetic lineages and recovered from different sources were hypothesized to produce homogeneous biofilm types. We tested this hypothesis using chi-square test. There was a significant difference between biofilm matrix phenotype in the two *S. saprophyticus* genetic lineages. In particular, biofilm composed of mainly PDS was strongly associated with lineage G (lineage G, 44%; 18/41; lineage S, 27%; 6/22, *p* < 0.0436), whereas protein-based biofilm was linked to isolates belonging to lineage S (lineage S, 50%; 11/22, lineage G, 32%; 13/41, *p* < 0.0468; Figure 2A).

Similarly, the protein-based phenotype that was lineage-linked was almost exclusive in human infection isolates (infection, 55%; 23/42, environmental sources, 5%; 1/21, *p* < 0.0001). Conversely, PDS-based biofilm was strongly associated with isolates from environmental sources including colonization and those of food origin (environmental sources, 71%; 15/21, infection, 21%; 9/42, *p* < 0.0001; Figure 2B). These results suggest that biofilm matrix phenotypes in *S. saprophyticus* vary not only according to the genetic background but also with the source of the isolates.

### Biofilm Phenotypes Are Not Associated With a Specific Genetic Content

**FIGURE 3:**
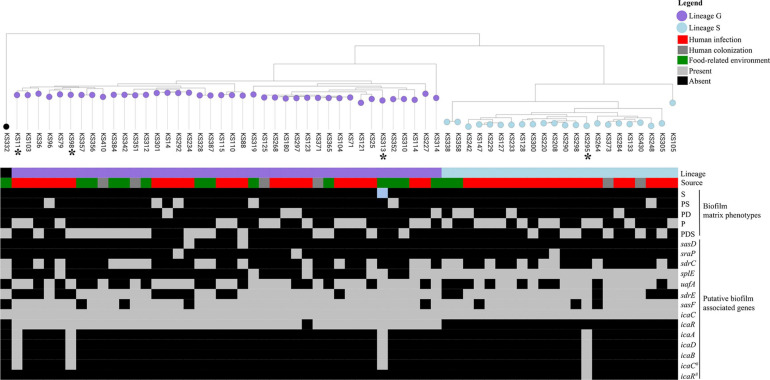
Maximum likelihood tree of 63 *Staphylococcus saprophyticus* strains showing the source, genetic lineages, matrix components of biofilm, and distribution of virulence genes. Distribution of five main biofilm matrix phenotypes produced by *S. saprophyticus* including S, polysaccharide; PS, protein–polysaccharide; PD, protein–eDNA; P, protein; and PDS, protein–eDNA–polysaccharide is shown. Isolates marked with asterisks carried additional *ica* genes (*icaA*, *icaD*, and/or *icaB*). Each node represents different strains, and nodes with the same color belonged to the same lineage. The tree was constructed from the core-genome single-nucleotide polymorphism (SNP) alignment without recombination by RAxML using general time reversible model and 100 bootstrap values for node support. A comparison figure was generated using microreact.

To investigate the frequency and distribution of biofilm-associated genes among the different biofilm matrix phenotypes produced by *S. saprophyticus*, we constructed a pangenome of the 63 isolates in the representative collection and assessed the prevalence of these genes (Jefferson, 2004; Rohde et al., 2007; Speziale et al., 2014; Barros et al., 2015; Fagerlund et al., 2016; Harris et al., 2016) in the accessory genome. Genes encoding autolysin (*atl*), fibronectin binding protein (*aas*), lipase (*ssp*), and elastin binding protein (*ebpS*) were present in all the isolates, while other genes encoding surface proteins, namely, *sasF*, *uafA*, and *sasD*, were present in 85, 56, and 3%, respectively. Serine protease-encoding genes *sdrH*, *sdrE*, *splE*, and *sdrC* were present in 66, 44, 39, and 5%, respectively. Additionally, *icaC* was present in 100% and *icaR* in 59% of the population tested. Complete *ica* gene cluster (*icaADBCR*) was only present in three infection isolates, while one other isolate recovered from a food-related environment carried *icaADBC* without *icaR* (Figure 3).

To better understand if any biofilm-associated genes or other genes in the genome were associated with specific biofilm phenotypes, we used pan-GWAS approach with Scoary where the five phenotypes based on the major matrix components were used as the predefined traits and mapped against the accessory genome containing 4,657 genes. However, we could not find a direct association between any gene and the matrix phenotypes produced by *S. saprophyticus* strains in this collection or the presence of specific components, such polysaccharide, protein, or eDNA. In spite of the fact that the great majority of strains produced biofilms containing polysaccharides, the complete *ica* cluster was only found in four isolates except one without *icaR*, suggesting that polysaccharide biofilms produced by *S. saprophyticus* are *ica* cluster independent.

### The Complete *icaADBCR* Cluster Was Acquired Multiple Times in *Staphylococcus saprophyticus*

In this study, we did not detect definite candidate genes associated with the biofilm formation ability or matrix heterogeneity observed in isolates analyzed. However, the observation that some *ica* genes were present in the 63 representative *S. saprophyticus* strains prompted a further assessment of the frequency and diversity of this gene cluster in the entire collection of 422 *S. saprophyticus* for which the genomic data were available. The draft genomes were annotated, the pangenome was constructed, and the prevalence of *ica* genes was assessed.

The *icaR* gene encoding a negative transcriptional regulator of the *ica* operon (Conlon et al., 2002) was exclusively found in isolates belonging to clonal lineage G, whereas an allele of *icaC* (*icaC_1*), a gene encoding an acetyltransferase that exports polysaccharide (Arciola et al., 2015), was ubiquitous in *S. saprophyticus*. The remaining *ica* genes, which are part of the cluster, namely, *icaA*, *icaD*, and *icaB*, were found in a very small fraction of the population (4/422; <2%). Three of the isolates containing the complete *ica* cluster belonged to clonal lineage G, while a single isolate belonged to clonal lineage S (see Figure 3).

To understand the relative position of the five *ica* genes and to estimate their location in the chromosome, we aligned and reordered the contigs against the reference genome *S. saprophyticus* 15305 and annotated the contigs containing *ica* genes. The ubiquitous *icaC* (*icaC_1*) and *icaR* genes were in different chromosomal regions; however, both were located in the first quarter of the genome (ATCC 15305 AP008934.1; *icaC_1*: 336965…338035; *icaR*: 134063…134569; Kuroda et al., 2005).

**FIGURE 4:**
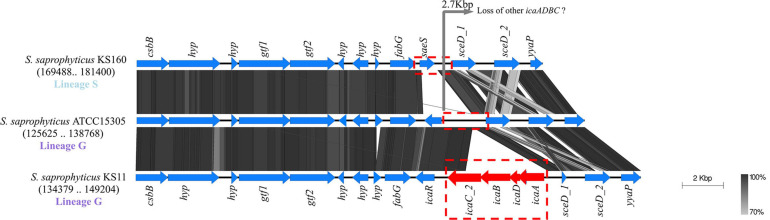
Evidence for probable loss of *ica* gene cluster in *Staphylococcus saprophyticus*. Blocks of identical color represent nucleotide sequence homology between regions found in the two species. The darker-shade region depicts the highest homology, and genes are represented by arrows facing the direction of transcription. The *icaR* gene was ubiquitous in lineage G; and within this lineage, additional *ica* genes that were found in some of the isolates were located downstream. *saeS* gene was located in place of *icaR* in lineage S. Comparison figure was generated using EasyFig.

The *icaADB* was located in three different chromosomal regions, always accompanied by an additional copy of *icaC* (*icaC_2*, *icaC_3*) different from the ubiquitous allele (*icaC_1*). In isolates belonging to lineage G, they were either located immediately downstream *icaR* or downstream *fmhA* nearby a *tRNA-Asn* (Figure 4 and Supplementary Figure 4).

**FIGURE 5:**
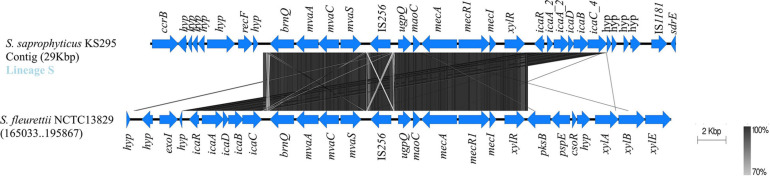
Evidence for the probable acquisition of *ica* gene cluster and SCC*mec*-associated genes in *Staphylococcus saprophyticus*. Structure of *ica* operon located downstream of SCC*mec* elements that were found in one *S. saprophyticus* isolate in lineage S. Blocks of identical color represent nucleotide sequence homology between regions found in the two species. The darker-shade region depicts the highest homology, and genes are represented by arrows facing the direction of transcription. The *ica* genes and the SCC*mec*-associated genes and the vicinity found in *S. saprophyticus* KS295 were compared with the closed genome of *Staphylococcus fleurettii* NCTC13829. Comparison figure was generated using EasyFig.

The single isolate from lineage S that contained an entire *ica* cluster carried these genes immediately downstream from the *mec* complex and within a genomic fragment bracketed by two different insertion sequences (IS*256* and IS*1181*). Although this *ica*-containing genomic region in *S. saprophyticus* resembles a SCC*mec*-like structure, due to the presence of the two central structural elements of SCC*mec* (*mec* complex A and *ccrB* gene; Figure 5), we could not find the inverted repeat regions and *IS*s defining the boundaries of the element. These elements appear to be inserted in a location far apart from the characteristic *orfX* chromosomal integration site (>1.5 Mb apart; see Figure 5; Rolo et al., 2017). Our results suggest that the complete *ica* cluster was acquired multiple times by *S. saprophyticus* in diverse chromosomal locations.

### The *ica* Cluster From *Staphylococcus saprophyticus* Originated From Other Coagulase-Negative Staphylococci

The diverse *ica* cluster genetic environment in *S. saprophyticus* suggests a complex evolutionary history for this group of genes. To better understand the origin and evolution of the cluster in *S. saprophyticus*, we used blast analysis (Altschul et al., 1997, 2005) to find homologs of these genes and compared the gene environment with those of related species.

The ubiquitous *icaC_1* and *icaR* from lineage G in *S. saprophyticus* was closely related homologous to the one found in *Staphylococcus xylosus* and *Staphylococcus equorum* (nt id: *icaC_1*—87% and 84%; *icaR*—72% and 62%, respectively). Furthermore, the genetic environment of ubiquitous *icaC_1* in both lineages (G and S) was very similar in terms of nucleotide sequence (nt id ∼ 80%) and gene synteny to the *icaC* region of *S. xylosus* and *S. equorum*, although it was inverted in these species (Supplementary Figure 3 and Table 1). The fact that *S. xylosus* and *S. equorum* are close phylogenetic relatives of *S. saprophyticus* and *icaC* genes are within a similar chromosomal region implies that they could have been inherited via vertical evolution during speciation. Actually, the average nucleotide identity between these species was identical (∼80%) to the homology observed for the genes understudy, which further supports the hypothesis of vertical inheritance.

**TABLE 1:** *ica* genes carried by four *Staphylococcus saprophyticus* strains in lineage G and S and their nucleotide homology with those found in other staphylococcal species.

Gene allele	Strains (lineage)	% Nucleotide identity					
		*Staphylococcus aureus*	*Staphylococcus xylosus*	*Staphylococcus cohnii*	*Staphylococcus fleurettii*	*Staphylococcus sciuri*	*Staphylococcus epidermidis*
*icaC_1*	G (326); S (*n* = 95)	56.0	**87.0**	55.0	53.0	54.0	56.0
*icaR*	All (G)	61.0	**75.0**	–	56.0	56.0	61.0
*icaA (group_4834)*	KS11, KS98 (G)	71.0	**78.0**	41.0	46.0	70.0	71.0
*icaD*		61.0	**74.0**	–	60.0	59.0	61.0
*icaB (group_4832)*		64.0	**73.0**	68.0	63.0	48.0	64.0
*icaC_2 (group_3085)*		69.0	**73.0**	63.0	54.0	56.0	50.0
*icaA_1*	KS313 (G)	71.0	78.0	**99.6**	67.0	70.0	71.0
*icaD_1(group_4346)*		60.0	64.0	**98.0**	59.0	58.0	59.0
*icaB_1*		70.0	73.0	**99.0**	63.0	62.0	66.0
*icaC_3 (group_2023)*		67.0	71.0	**99.0**	63.0	61.0	68.0
*icaR_1*	KS295 (S)	75.0	56.0	–	**99.8**	73.0	56.0
*icaA_2*		83.0	70.0	65.0	**99.8**	83.0	65.0
*icaD_2*		80.0	60.0	–	**99.7**	77.0	56.0
*icaB_2*		77.0	65.0	64.0	**100.0**	78.0	64.0
*icaC_4*		79.0	59.0	58.0	**99.8**	73.0	52.0

*depicts genes that either were completely absent or had ≤60% coverage. Values highlighted in bold had the highest identities for each gene.*

*Staphylococcus xylosus* is one of the species most closely related to *S. saprophyticus* in the *Staphylococcus* phylogenetic tree (Naushad et al., 2019) and also contains *icaC_1* ubiquitously and *icaR* in low frequency (*n* = 16/57) in their genome, suggesting that these genes could have been transmitted during vertical evolution through speciation. However, the extremely low GC content observed in these two genes in both *S. saprophyticus* and *S. xylosus* (*icaC*: 28.85, 30.81%; *icaR*: 25.9, 27.84%) when compared with the remaining genome implies that they were acquired from other genus into *Staphylococcus*.

Among the four *ica*-positive strains from lineage G, two carried *icaADBC* (*icaA*, *icaD*, *icaB*, and *icaC_2*; KS11 and KS98, Figure 3) located downstream the ubiquitous *icaR* (Figure 4). Like with ubiquitous *icaR*, the remaining *ica* genes (*icaADBC*) had the closest homology with those of *S. xylosus* (nt id = 73–78%; Table 1). The gene organization within the clusters was similar to the *ica* cluster from *Staphylococcus aureus* or *S. epidermidis* (Lerch et al., 2019). The only exception was that the *icaR* had the same transcriptional direction as the remaining *ica* genes (Figure 4).

The other two *ica*-positive strains from lineage G that carried *icaADBC* downstream *fmhA* gene appear to have a completely different origin. They contained no *icaR* (*icaADBCΔR*); and all the other *ica* genes (*icaA_1*, *icaB_1*, *icaD_1*, and *icaC_3*) were highly similar (nt id ≥ 98%) to those in *Staphylococcus cohnii* BKAW01 (Table 1 and Supplementary Figure 4). The high similarity of *ica* genes detected in these strains with those of *S. cohnii* suggests a probable recent acquisition from this species. In fact, genes encoding a *tRNA* [*tRNA-Asn (att)*], a putative recombinase (*bin*), and a plasmid replication protein (*rep*), all genes associated with mobilization and recombination, were located downstream of the *ica* cluster, suggesting that these genes might have been exogenously acquired.

In the single isolate from lineage S that contained an entire *ica* cluster (*icaA_2*, *icaD_2*, *icaB_2*, *icaC_4*, and *icaR_1*) in a SCC*mec*-like structure (KS295; Figures 3, 5), the *ica* cluster genes were almost identical (nt. Id ≥ 99.7%) to those found in *Staphylococcus fleurettii* having a much lower identity with *ica* genes than other staphylococcal species (*S. aureus*, 75–83%; *S. epidermidis*, ≤67%; *Staphylococcus sciuri*, 73–83%; and *S. xylosus*, ≤70%; Table 1). This is the only case in which direction of transcription of *icaR* is opposite to the remaining *ica* genes, as it is described for *S. aureus* and *S. epidermidis* (Lerch et al., 2019). Interestingly, in addition to *ica* cluster, the entire region spanning from *brnQ* to *xylR*, including the *mec* complex, was highly identical to the *mecA* region in *S. fleurettii* NCTC13829 (99–100% nt id; Table 2 and Figure 5). Furthermore, the gene synteny of this region in both *S. saprophyticus* and *S. fleurettii* NCTC13829 was similar. The only exception was the position of *ica* gene cluster that was found upstream of the *mec* complex and SCC*mec* elements in *S. saprophyticus* and downstream these elements in *S. fleurettii* (Figure 5). The chromosomal location of the *mec* complex in *S. fleurettii*, so-called the native location (approximately 200 kb apart from *orfX*; Rolo et al., 2017), differed with the location of *mec* complex and *ica* genes in this *S. saprophyticus* strain. Overall, our data suggest that *ica* genes in *S. saprophyticus* were probably acquired from different CoNS species and were inserted in different chromosomal locations.

**TABLE 2:** SCC*mec*-associated genes carried by a *Staphylococcus saprophyticus* strain (KS295) in lineage S and their homology with those found in other staphylococcal species.

Genes	% Nucleotide identity			
	*Staphylococcus aureus*	*Staphylococcus fleurettii*	*Staphylococcus sciuri*	*Staphylococcus vitulinus*
*ccrB (ccrB3)*	86.0	–	**91.0**	–
*brnQ*	74.0	**99.9**	83.0	86.0
*mvaA*	70.0	**100.0**	83.0	87.0
*paaJ*	–	**99.9**	85.0	85.0
*mvaS*	99.7	**99.9**	88.0	91.0
IS*256*	–	**99.7**	79.0	–
*ugpQ*	**100.0**	–	–	–
*maoC*	**100.0**	–	–	–
*mecA*	99.9	**100**	99.9	99.9
*mecR1*	99.5	**99.7**	99.5	–
*mecI*	**100.0**	**100.0**	–	–
*xylR*	99.0	**99.8**	99.0	–

*depicts genes that either were completely absent or had ≤60% coverage. Values highlighted in bold had the highest identities for each gene.*

## Discussion

In this study, we confirmed that almost all (91%) the 422 included *S. saprophyticus* isolates produced biofilm irrespective of the source or genetic lineage of the isolates. This rate was higher than that found in a previous study wherein 70% (119/169) of the *S. saprophyticus* recovered from infection and food-produced biofilm (Martins et al., 2019). These high rates of biofilm formation in the population suggest that biofilm is probably the main mode of living of these bacteria. Biofilm formation is an important step in bacterial colonization and adaptation in a variety of environments and contributes to disease development in the host (Speziale et al., 2014; Jeong et al., 2016). Biofilm formation in this study was assessed using the *in vitro* microtiter plate assay in rich standard medium. This does not completely mimic the *in vivo* scenario; and, hence there is the possibility for false negatives. Studies on comparison of different techniques would be essential to complement the results obtained here.

The structure and composition of biofilms produced by bacteria are generally maintained by various macromolecules, which can vary in type and quantity among species and strains (Arciola et al., 2015; Sugimoto et al., 2018). The *S. saprophyticus* biofilm matrix composition determined by detachment assays in this study was highly heterogeneous, showing at least five different phenotypes. However, the most common biofilm types found in this study were composed of only protein or PDSs. Noteworthy, we found significant differences in biofilm matrix composition in *S. saprophyticus* of the two genetic lineages as well as between strains of infection and colonization/environmental origin (lineage S/infection: protein; lineage G/colonization: PDS). The heterogeneity in the biofilm matrix phenotypes found in *S. saprophyticus* population was previously described for *Staphylococcus aureus* (Sugimoto et al., 2018) and *S. epidermidis* (Rohde et al., 2007), among others. Moreover, Tasse and colleagues have previously described the association between biofilm matrix phenotypes and clonal lineages in *S. aureus*. In particular, they reported that *S. aureus* CC5, CC15, and CC30 strains produced highly eDNA-dependent biofilm, whereas that *S. aureus* CC45 was protein-dependent (Tasse et al., 2018). This observation might be a long-term adaptive response to different environmental signals that may vary between different settings, such as the presence of antibiotics, immune system, acidity, humidity, changes in temperature, and other imbalances in the environment that may induce stress (Rachid et al., 2000; López et al., 2010; Di Ciccio et al., 2015). Results suggest that eradication of biofilms in infection and colonization should be done with different approaches. More knowledge on the composition and genetic basis of biofilm formation is important to help develop anti-biofilm strategies against this pathogen.

Genes that encode cell wall-anchored proteins, surface proteins, autolysins, and *ica* operon that are linked to biofilm formation and matrix phenotypes were found in our collection. Adhesin-encoding genes such as *aas* (Hell et al., 1998), *ebpS* (Downer et al., 2002), and *uafA* (Kuroda et al., 2005) had been speculatively linked to biofilm formation in *S. saprophyticus* with scarce experimental proof (Fagerlund et al., 2016). In this study, the presence of these genes alone was not correlated with biofilm formation ability or composition, suggesting that mutations with impact in gene expression level might have an important role on biofilm phenotype produced, as previously described (Harris et al., 2016; Tasse et al., 2018). Further studies would be required to confirm this hypothesis. Another possibility is that the low number of isolates included in the GWAS analysis could have hindered the identification of statistically significant associations.

Among the genes associated with biofilm formation in staphylococci, the *ica* operon, responsible for the production of polysaccharide, is the most important (Rohde et al., 2010). However, in our *S. saprophyticus* collection, this operon was rarely found (∼1%), suggesting that biofilm in this species is *ica*-independent. Although the complete cluster was scarce, an allele of *icaC* gene, involved in polysaccharide export, was found to be ubiquitous (Arciola et al., 2015), and *icaR*, a *ica* negative transcriptional regulator (Conlon et al., 2002), was present in all isolates of lineage G. In spite of their high homology with genes within the *ica* cluster, these two genes, when together in the same strain, were located far apart in the chromosome. These genes had high similarity (nt id ∼ 80%) with those found in *S. xylosus*, a close phylogenetic relative of *S. saprophyticus*, within a similar chromosomal region, implying that they could have been inherited via vertical evolution during speciation.

Besides the ubiquitous *icaC*/*icaR* genes, we additionally found in the genome of a few strains the complete *ica* cluster located in three different regions of the chromosome: downstream, the ubiquitous *icaR* or nearby genes usually associated with mobile genetic elements like SCC*mec* or plasmids. While *ica* cluster genes located nearby the ubiquitous *icaR* had a low identity with *ica* genes from the closely related species *S. xylosus* (∼70% nt id), *ica* cluster genes inserted nearby mobile genetic elements showed a high identity (>98%) with *ica* genes from other coagulase-negative staphylococci such as *S. cohnii* and *S. fleurettii*. These results suggest that the *ica* cluster located downstream *icaR* was either acquired exogenously, a long time ago, or inherited via vertical evolution during speciation and further lost from the majority of the population to avoid the “fitness cost” associated with polysaccharide production. On the other hand, the other *ica* clusters identified appear to have been acquired by horizontal gene transfer from staphylococcal species.

## Conclusion

In this study, we showed that there was a high variability in the composition of the biofilm formed by *S. saprophyticus*. The most common type of biofilm produced by this bacterium contained protein or PDSs. The biofilm components appear to differ between food-related and human infection isolates and between clones, suggesting that modulation of biofilm composition could be a key step in *S. saprophyticus* virulence and niche adaptation. Our data further showed the possible origin and multiple acquisition of the *ica* gene cluster in *S. saprophyticus*.

## Data Availability Statement

The datasets presented in this study can be found in online repositories that can be found in the article/Supplementary Material. Sequence data generated from this study had earlier been deposited to the Sequence Reads Archives under the project accession number PRJNA604222.

## Ethics Statement

The studies involving human participants were reviewed and approved by Comissão de Ética para a Investigação e Ensino (CEIE) da Faculdade de Medicina Veterinária, Universidade de Lisboa. Written informed consent for participation was not required for this study in accordance with the national legislation and the institutional requirements. The animal study was reviewed and approved by Comissão de Ética para a Investigação e Ensino (CEIE) da Faculdade de Medicina Veterinária, Universidade de Lisboa. Written informed consent for participation was not obtained from the owners because Oral informed consent was obained for animal participation. Slaughterhouse samples were part of the routine control practices for evaluation of good hygiene practices and programs to assure meat safety (CE No. 853/2004). The sampling was done as part of the routine infection control program and no additional sampling was perfomed. The samples were non-invasive or minimal invasive and were the animal commensal bacteria and not animals that were studied.

## Author Contributions

OL and MB performed the phenotypic experiments. OL performed the bioinformatics analysis. OL and MM carried out the data analysis and interpretation and wrote the manuscript. MF, LG, PP, EG, CT, JE, MU, HL, HW, and MDB provided the isolates. MF, LG, PP, EG, CT, JE, MU, HL, HW, PW, and MDB were involved in manuscript revision. All authors read and approved the final manuscript.

## Conflict of Interest

The authors declare that the research was conducted in the absence of any commercial or financial relationships that could be construed as a potential conflict of interest.

## Footnotes

1 http://www.ecdc.europa.eu/en/activities/surveillance/EARS-Net/Pages/index.aspx

## Supplementary Material

Supplementary Figure 1 Maximum likelihood tree of 422 *S. saprophyticus* constructed with core-genome single nucleotide polymorphism (SNP) highlighting the selected strains in the population. Each node represents different strains and node of the same color belonged to the same lineage. The core genome alignment was constructed using CSI-Phylogeny. Recombination regions were removed using Gubbins and the phylogenetic tree reconstructed using RAxML with general time reversible model and 100 bootstrap value for node support.

Supplementary Figure 2 **(A)** Biofilm present after treatment with biofilm degrading agents in 63 *S. saprophyticus* strains. Data presented are means and standard errors of the present biofilm after treatment compared to control (sodium acetate) expressed in percentage. Assays were carried out in triplicates. **(B)** Biofilm present after treatment with biofilm degrading agents for 63 *S. saprophyticus* strains. Data presented are means and standard errors after treatment compared to control (sodium acetate) measured at OD595 nm. Assays were carried out in triplicates.

Supplementary Figure 3 Comparison of *icaC* environment in *S. saprophyticus*, *S. xylosus*, and *S. equorum*. Blocks of identical color represent nucleotide sequence homology between regions found in the two species. Darker shade colored region depicts highest homology and genes are represented by arrows facing the direction of transcription. IcaC and its vicinity found in *S. saprophyticus* lineages were compared with each other and with that of *S. saprophyticus* ATCC 15305, as well as with the closed genome of the phylogenetic relative species of this bacterium. Comparison figure was generated using EasyFig.

Supplementary Figure 4 Structure and comparison of *ica* gene cluster found in a *S. saprophyticus* recovered from food-related environment and belonging to lineage G. Blocks of identical color represent nucleotide sequence homology between regions found in the two species. Darker shade colored region depicts highest homology and genes are represented by arrows facing the direction of transcription. All the *ica* genes including some of the genes in its vicinity are highly similar (≥98% nucleotide sequence) with those found in *S. cohnii.* The vicinity of the *ica* genes was compared with the draft genome of *S. cohnii* BKAW01. Comparison figure was generated using EasyFig.

Supplementary Table 1 Characteristics of *S. saprophyticus* used in this study.

Supplementary Table 2 Characteristics of *S. saprophyticus* isolates used for the biofilm detachment assay.
