# Supplementary figures and images for: Staphylococcus saprophyticus From Clinical and Environmental Origins Have Distinct Biofilm Composition
Source: Front Microbiol. 2021 Jun 7;12:663768. doi: 10.3389/fmicb.2021.663768 (PMC8216562; doi:10.3389/fmicb.2021.663768)

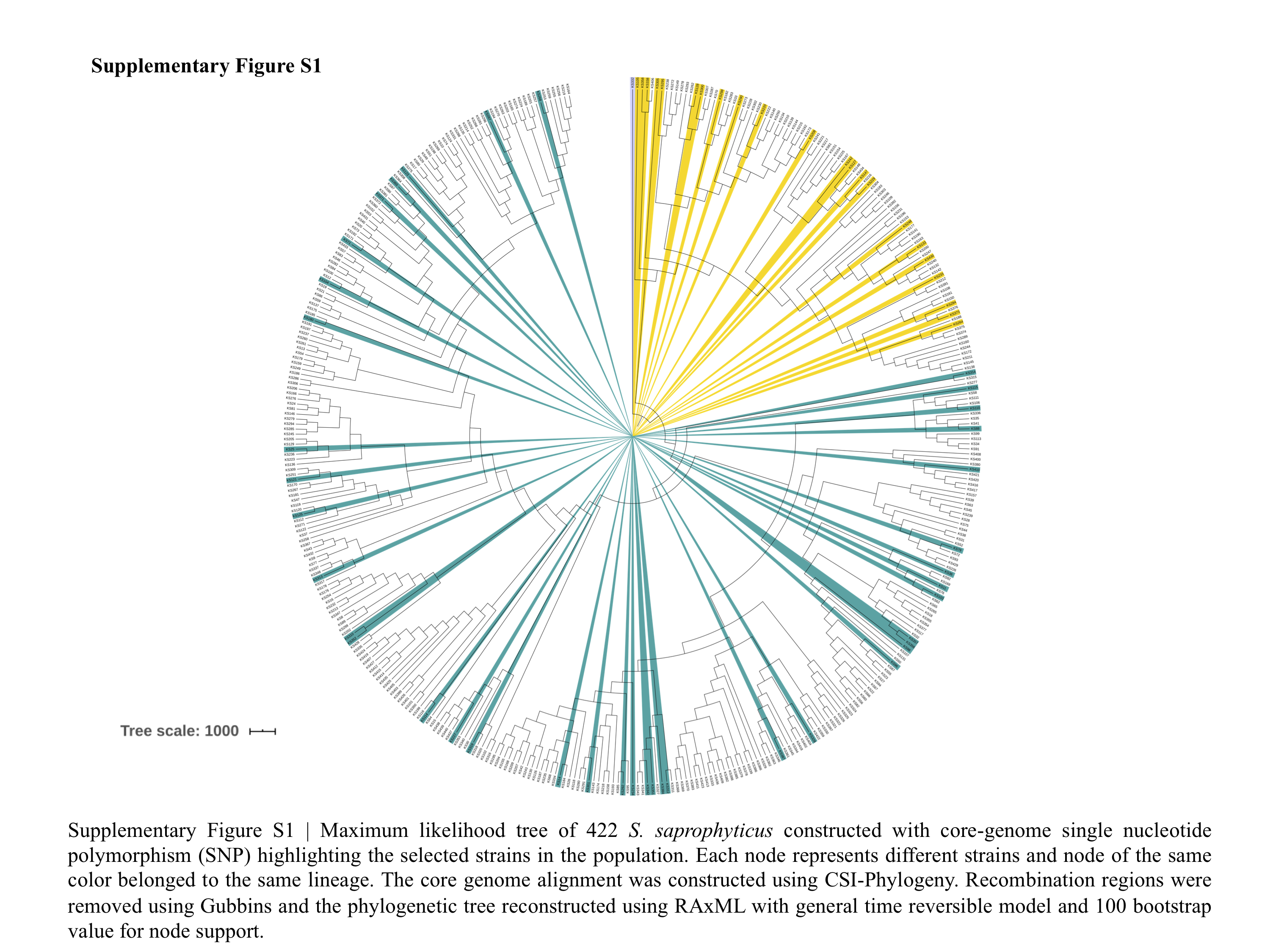

Supplement: Supplementary Figure 1 — Maximum likelihood tree of 422 S. saprophyticus constructed with core-genome single nucleotide polymorphism (SNP) highlighting the selected strains in the population. Each node represents different strains and node of the same color belonged to the same lineage. The core genome alignment was constructed using CSI-Phylogeny. Recombination regions were removed using Gubbins and the phylogenetic tree reconstructed using RAxML with general time reversible model and 100 bootstrap value for node support. [file Image_1.tiff]

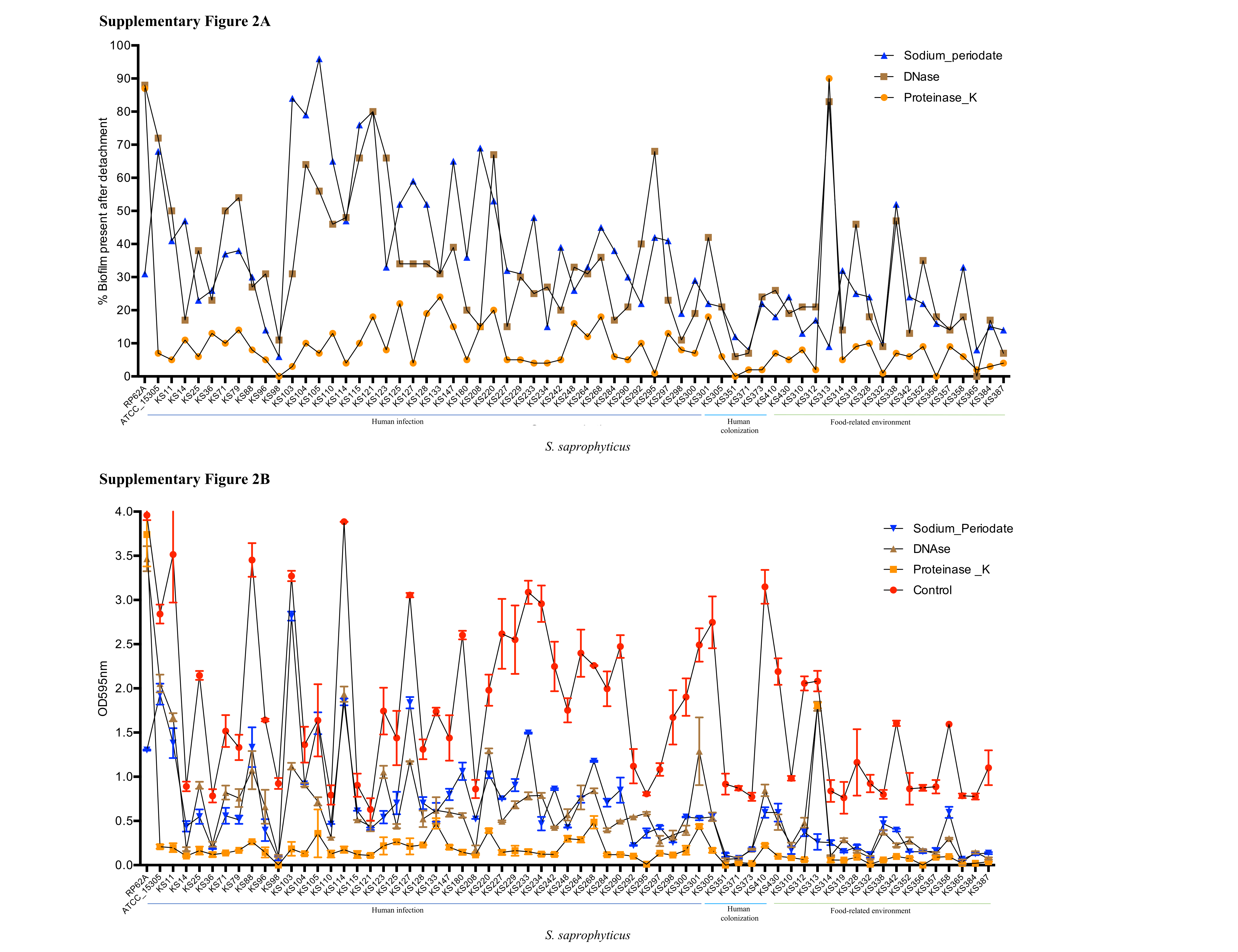

Supplement: Supplementary Figure 2 — (A) Biofilm present after treatment with biofilm degrading agents in 63 S. saprophyticus strains. Data presented are means and standard errors of the present biofilm after treatment compared to control (sodium acetate) expressed in percentage. Assays were carried out in triplicates. (B) Biofilm present after treatment with biofilm degrading agents for 63 S. saprophyticus strains. Data presented are means and standard errors after treatment compared to control (sodium acetate) measured at OD595 nm. Assays were carried out in triplicates. [file Image_2.tiff]

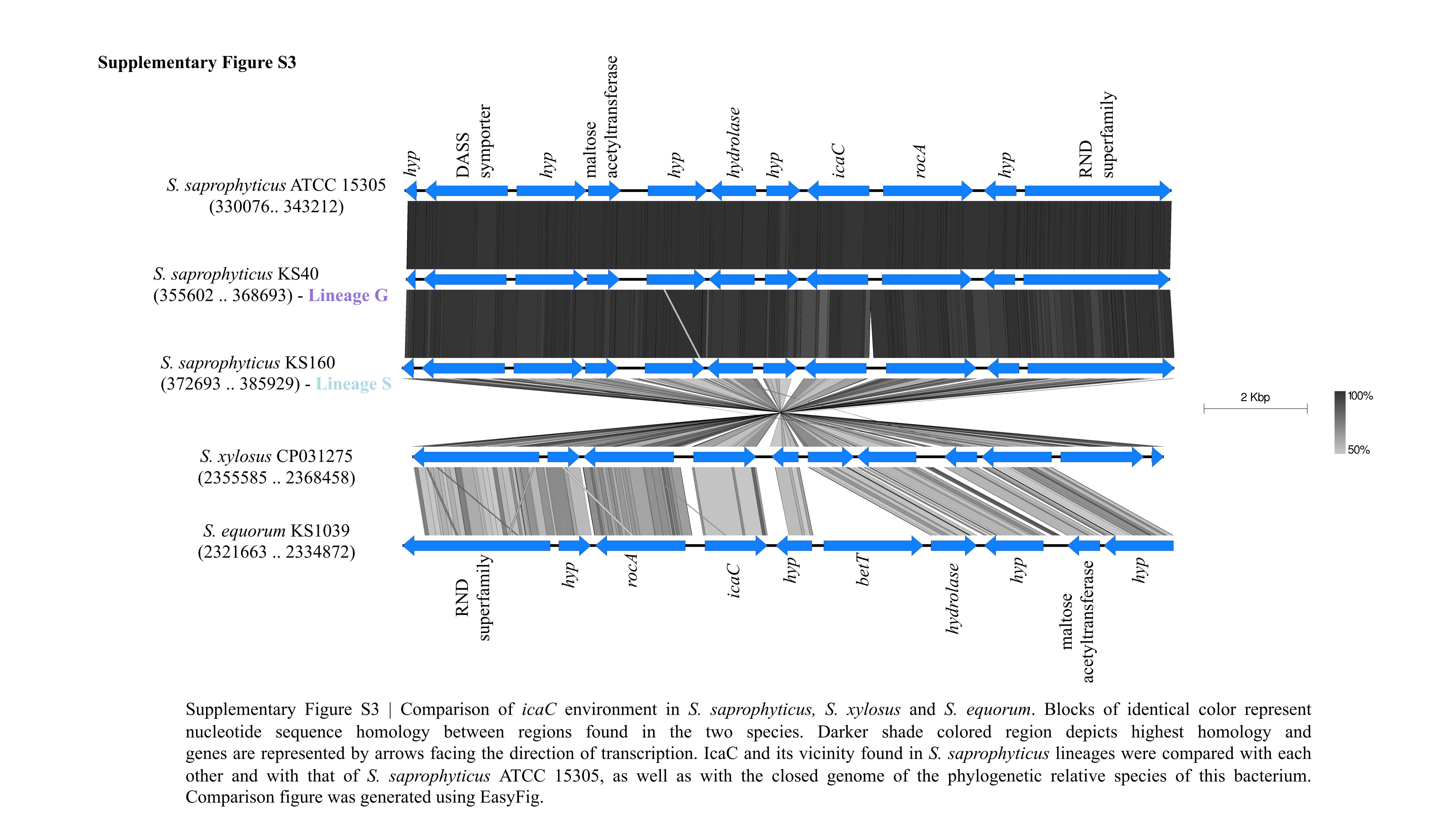

Supplement: Supplementary Figure 3 — Comparison of icaC environment in S. saprophyticus, S. xylosus, and S. equorum. Blocks of identical color represent nucleotide sequence homology between regions found in the two species. Darker shade colored region depicts highest homology and genes are represented by arrows facing the direction of transcription. IcaC and its vicinity found in S. saprophyticus lineages were compared with each other and with that of S. saprophyticus ATCC 15305, as well as with the closed genome of the phylogenetic relative species of this bacterium. Comparison figure was generated using EasyFig. [file Image_3.tiff]

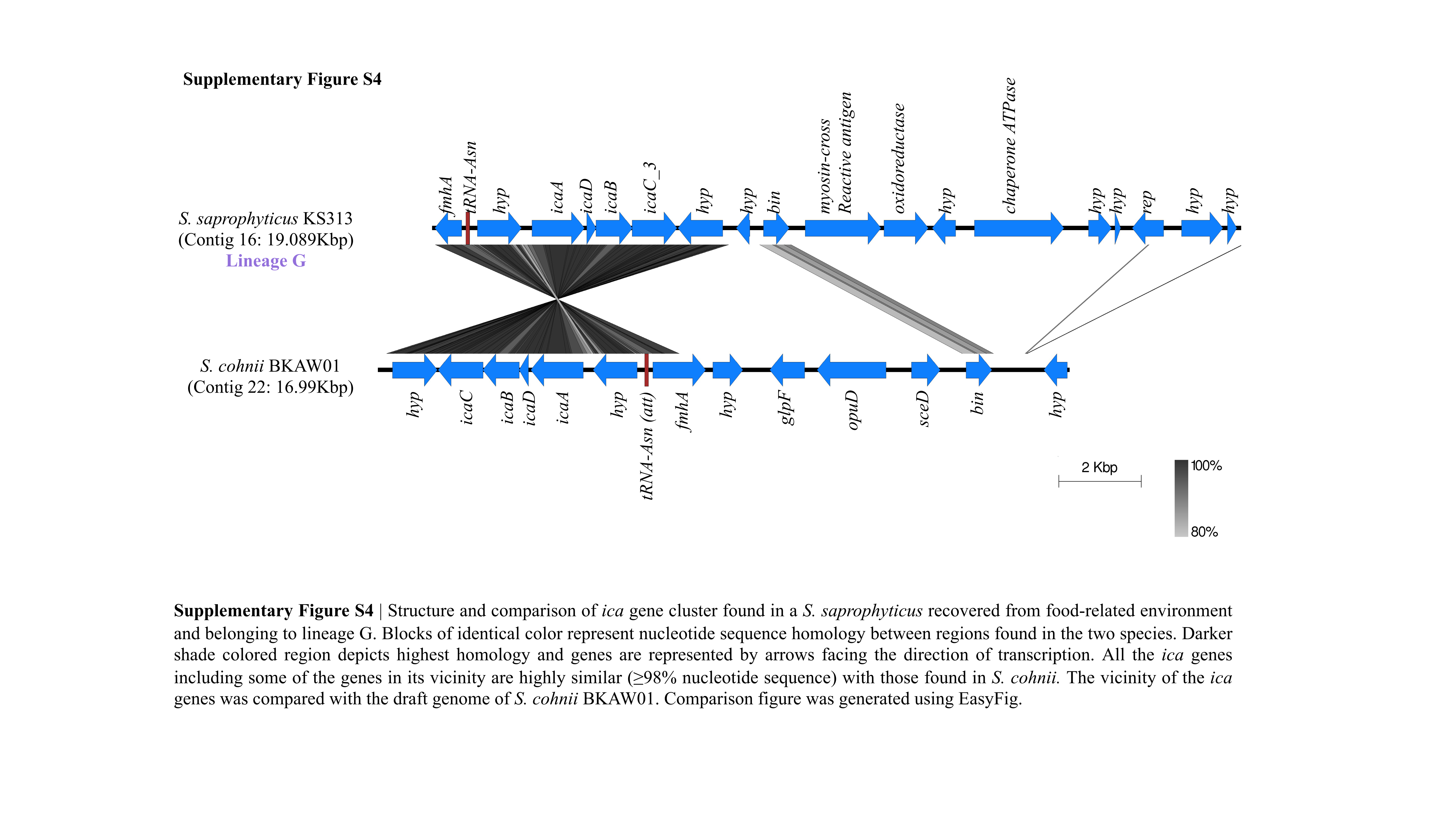

Supplement: Supplementary Figure 4 — Structure and comparison of ica gene cluster found in a S. saprophyticus recovered from food-related environment and belonging to lineage G. Blocks of identical color represent nucleotide sequence homology between regions found in the two species. Darker shade colored region depicts highest homology and genes are represented by arrows facing the direction of transcription. All the ica genes including some of the genes in its vicinity are highly similar (≥98% nucleotide sequence) with those found in S. cohnii. The vicinity of the ica genes was compared with the draft genome of S. cohnii BKAW01. Comparison figure was generated using EasyFig. [file Image_4.tiff]
